# Are radiology residents safe to report feeding nasogastric (NG) tubes on chest X-rays?

**DOI:** 10.1093/bjro/tzag001

**Published:** 2026-01-10

**Authors:** Cindy Chew, Lucy McGuire, Patrick J O’Dwyer, David Young

**Affiliations:** Undergraduate School of Medicine, University of Glasgow, University Avenue, Glasgow, G12 8QQ, United Kingdom; Department of Radiology, University Hospital Hairmyres, NHS Lanarkshire, Eaglesham Road, East Kilbride, G75 8RG, United Kingdom; Undergraduate School of Medicine, University of Glasgow, University Avenue, Glasgow, G12 8QQ, United Kingdom; Undergraduate School of Medicine, University of Glasgow, University Avenue, Glasgow, G12 8QQ, United Kingdom; Department of Mathematics and Statistics, University of Strathclyde, 26 Richmond Street, Glasgow, G1 1XH, United Kingdom

**Keywords:** NG tube, patient safety, never event, competency training, resident medical education

## Abstract

**Objectives:**

The task of issuing reports on whether nasogastric (NG) tubes are safe for enteral nutrition on chest X-ray (CXR) often falls to radiology residents. The aims of this study are to evaluate whether radiology residents are formally trained and their performance in interpreting NG tube position on CXR.

**Methods:**

Radiology residents were invited to participate in an online study evaluating NG tube position on CXR. The CXR images comprised 20 NG tubes, 14 of which were correctly sited, while 4 were in the distal oesophagus and 2 in the lung.

**Results:**

Twenty-eight (of 185, 15%) radiology residents responded—despite incentives to participate and directed by Training Program Directors/Heads of School. Of those, only 10 (35.7%) correctly identified all NG tube positions on CXR. The most common error was reporting a *correctly sited* NG tube as mal-positioned for enteral nutrition. Global error rate was 8.9%. Radiology residents who correctly interpreted all 20 NG tube CXRs were significantly more confident in their abilities on a 5-point Likert scale than those who got at least 1 NG tube CXR wrong [4.4 (0.52) versus 3.8 (0.79), *P* = .02].

**Conclusions:**

This study suggests that radiology residents may not be adequately trained to interpret the position of NG tubes on CXRs. Early and compulsory training in this important skill should be instituted urgently.

**Advances in knowledge:**

There is a critical gap in radiology training. Radiology residents may not be adequately prepared to safely interpret NG tube position on chest X-rays. New DHSC memorandum of understanding mandates competency-based education across all training programs.

## Introduction

Enteral nutrition is a critical therapy for patients unable to sustain adequate oral intake. It provides essential macro- and micronutrients and can be life-saving.^1^ Industry reported global enteral feeding tube market was valued at US$2.5 billion in 2022 and is projected to reach US$4.1 billion by 2030.^2^

Nasogastric (NG) tubes are widely used but carry a persistent risk of fatality if used inappropriately. Although generally safe, instances of administering nutrition through misplaced tubes remain a preventable complication associated with high mortality.^3^^,^^4^ A recent study showed that senior medical students were unable to reliably identify misplaced NG enteral feeding tubes, though this deficit was improved with training.^5^ In the National Health Service (NHS), administering enteral nutrition through misplaced NG tubes remains one of the most frequent “never events”, with 270 incidents between 2014 and 2023 and at least 21 annually over the past 5 years—despite repeated safety alerts and professional guidance.^6^^,^^7^

Technological solutions have not yet eliminated the problem. Multiple commercial devices and artificial intelligence (AI) tools have been developed to support NG tube placement, but none have demonstrated sufficient reliability for widespread adoption.^8^ Consequently, many hospitals mandate NG tube chest x-rays (CXR) be formally reported before commencing enteral nutrition.

Amid a 30% shortfall in clinical radiologists, this task often falls to radiology residents.^9^ Under Ionising Radiation (Medical Exposure) Regulations [IR(ME)R] 2017, these residents are the responsible practitioners who should undertake reporting only after adequate training.^10^ Patient Safety Alerts specify competency-based training—both theoretical and practical—followed by assessment. Gaps in training pathways may leave radiology residents potentially liable for “never events”.

To expedite care and enhance safety, a radiographer-led, competency-based training programme has recently been introduced.^11^ The rigour of such programmes should be assured to maintain patient safety. Meanwhile, an international study of diagnostic radiographers (*N* = 68) found that only 76% of NG tube CXRs were interpreted correctly, with a 3% error rate for “never events”.^12^

The aims of this study are to evaluate (1) whether radiology residents in Scotland receive formal training in interpreting NG tube position on CXR and (2) their diagnostic performance.

## Methods

Institutional review board approval for this study was sought but waived, as this was deemed a quality improvement exercise and audit. Consent was implied by participation and data were anonymous.

### Participants

All radiology resident doctors in Scotland (*N* = 185) were eligible to participate in the anonymous online CXR-NG tube position evaluation survey (March 7-May 27, 2024). Invitations to participate were sent by email from Training Program Directors. Participation was promoted at the Scottish Radiological Society Spring Meeting (May 3, 2024) and one reminder email was sent. All email communications were standardized. Participation was voluntary. A small monetary prize and being a named contributor to the work were offered to every participant to encourage uptake; named contribution was optional.

### The NG tube CXR test

**Figure 1.**
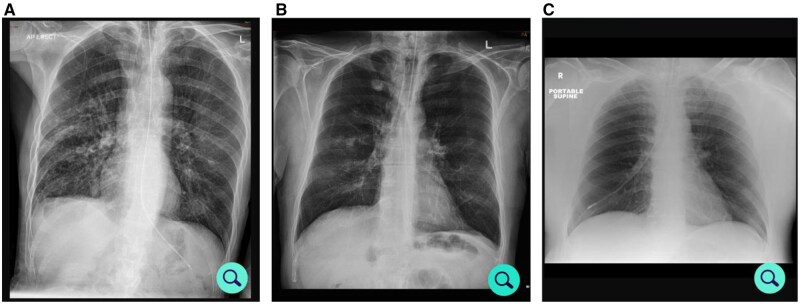
CXR images shown to respondents in which the NG Tube tip is correctly positioned in the stomach (A), mal-positioned in the oesophagus (B), or mal-positioned in the lung (C).

Twenty CXR images of NG tubes used in a previous study were deployed (online Google Form).^5^ These comprised 14 correctly sited (Figure 1A) and 6 mal-positioned NG tubes. The tips of 4 mal-positioned NG tubes were in the oesophagus (Figure 1B), while 2 were in the lung (Figure 1C). The number and case mix were decided following discussion with a senior statistician.

Participants were asked to evaluate each CXR on whether the NG tube position was correct for administering enteral nutrition (“Yes” or “No”). They were blinded to the number of correct/misplaced tubes. Sequence of CXRs was random and no time restriction was applied, time taken to complete the evaluation was not collected.

All 20 CXRs were anonymized, unannotated, performed with posterior-anterior (PA) projection, and included after independent review by 2 senior radiology consultants of over 10 years’ experience each. Assessors were blinded to each other’s reviews and CXRs were only selected when both were in agreement with the quality of the images. Further technical details below and available from our previous study.^5^

### NG tube CXR interpretation: training and self-rated confidence

All respondents were asked:

1. whether they were formally taught to interpret NG tube position on CXR
2. to rate their confidence in their ability to correctly interpret the position of an NG tube on a 5-point Likert scale (1—not confident; 5—very confident), and
3. if they would like more teaching/resources on how to read CXR for NG tube placements. Responses were collated and performance (scores) was evaluated (see Appendix S1).

### Statistics

Data are expressed as mean with standard deviation and confidence interval where appropriate. Differences in the number of NG tube CXRs correctly identified between the groups were assessed using chi-squared test. Differences in the confidence rating between the groups were assessed using 2-sample *t*-test. Analyses were performed using Minitab LLC (version 18) at a 5% significance level.

## Results

Twenty-eight of 185 (15%) radiology residents responded to the survey. Response rates varied across the training schemes, with Scheme A having a statistically significant lower response rate [7%, (chi-square test) *P* = .042] compared to the 3 other programs. All specialty-training years from the 4 training schemes in Scotland were represented. The majority of respondents (*N* = 20, 71.5%) gained their primary medical qualification in the United Kingdom. Twenty-two (78.6%) underwent Foundation training only prior to commencing their Radiology training (Table 1). Twenty-seven (96.4%) had passed the Fellow of the Royal College of Radiologists (FRCR) Part 1 Anatomy/Physics examination at the time of participation in this study.

**Table 1.** Radiology demographics (*N* = 28).

	Category	Number of respondents, *n* (%)
**1**	**Specialty training (ST) year**	
	ST 1	3 (10.7)
	ST 2	5 (17.9)
	ST 3	11 (39.3)
	ST 4	7 (25)
	ST 5	2 (7.1)
**2**	**Scottish training scheme^a^**	
	A	6 (21.4)
	B	9 (32.1)
	C	7 (25)
	D	6 (21.4)
**3**	**Primary medical qualification**	
	United Kingdom	20 (71.4)
	International	8 (28.6)
**4**	**Previous clinical training**	
	Foundation	22 (78.6)
	Core surgery	3 (10.7)
	Core medicine	2 (7.1)
	Other^b^	1 (3.6)
**5**	**Previous training in NG tube position on CXR**	
	Yes	12 (42.9)
	No	16 (57.1)

a Four schemes in Scotland (in no particular order) are: East, North, South East, and West.

b Respondent did not specify in text box provided.

Only 10 (35.7%) radiology residents correctly interpreted all 20 of the NG tube positions on CXR for enteral nutrition. Four (14.3%) radiology residents incorrectly identified NG tube tips at the gastroesophageal junction (GOJ) as suitable for enteral nutrition administration. The most common error was reporting a *correctly sited* NG tube as *mal-positioned* for enteral nutrition (18 radiology residents; 64.3%). This occurred on 46 occasions, giving a likelihood a radiology resident incorrectly interpreting the position of an NG tube on CXR (when GOJ cases were included) of 50 times out of 560 (the total number of CXRs viewed by 28 radiology residents) or 8.9%.

There was no difference in the number of radiology residents who correctly identified all 20 of the NG tubes on CXRs in relation to the year of radiology training, whether international or UK primary medically qualified, underwent previous post-graduate Core or NG tube CXR training. There was however a statistical difference between training schemes, with residents from Schemes A and B performing better than those from Schemes C or D (*P* = .031) (Table 2).

**Table 2.** Proportion of correct NG tube CXR responses by training schemes.

Training scheme	Perfect score (all 20 correct) *N* [%]	≥1 incorrect *N* [%]	Total respondents *N*
A	4 [66.7]	2 [33.3]	*6*
B	5 [55.6]	4 [44.4]	*9*
C	0 [0]	7 [100.0]	*7*
D	1 [16.7]	5 [83.3]	*6*
All	10 [35.7]	18 [64.3]	*28*

Chi-squared test *P* = .031.

### “Never Event” 

Of the 20 NG tube CXRs, 2 were incorrectly positioned within the lungs—so called “never event” CXRs for enteral nutrition delivery. Across the entire respondent group, there were 56 opportunities (from 28 participants each viewing 2 lung-placed CXRs) to identify a “never event”. All 28 radiology residents (100.0%) correctly interpreted these NG tubes as mal-positioned on CXR and should not be used to administer enteral nutrition.

### Training and self-rated confidence

Sixteen (57.1%) radiology residents reported receiving no formal training in the interpretation of NG tube position on CXR. These residents were across all training Schemes. The overall mean self-rated confidence for the group at interpreting NG tube position on CXR was high: 4.0 (0.74) on a 5-point Likert scale (5 being “very confident”). It was 3.83 (0.79) for those who had at least 1 NG tube CXR wrong (*N* = 18) compared to 4.4 (0.52) for those who correctly identified all 20 NG tube CXR positions (*P* = .025) (Table 3). Mean self-rated confidence of radiology residents who reported NG tubes mal-positioned at gastroesophageal junction as suitable for enteral nutrition was low at 3.3 (0.96) (Table 3).

**Table 3.** Self-rated confidence of radiology residents in NG tube CXR interpretation (5-point Likert scale).

Category	Mean self-rated confidence (mean SD)	95% CI
All (*N* = 28)	4.00 (0.74)	3.73-4.27
All correct (*N* = 10)^a^	4.40 (0.52)	4.08-4.72
≥1 incorrect (*N* = 18)^a^	3.83 (0.79)	3.47-4.19
Mal-positioned NG tube reported as safe for enteral nutrition (*N* = 4)	3.30 (0.96)	2.36-4.24

a *P* = .025 between those with perfect and imperfect scores.

Twenty-one radiology residents (75%) would like additional training in NG tube CXR evaluation—15 reported at least 1 NG tube CXR incorrectly while 6 had perfect scores (*P* = .17). All 4 who reported a misplaced NG tube at the gastroesophageal junction as safe for enteral nutrition would like further training, while of concern 3 (10.7%) radiology residents who misidentified at least 1 correctly sited NG tube as mal-positioned on CXR did not feel further training was required.

## Discussion

### Lack of standardized training

This study highlights a lack of consistent and standardized training in interpreting NG tube position on CXR across Radiology Training Programs in Scotland. This falls short of the National Patient Safety Alerts (2011, 2016), which mandate competency-based training for staff interpreting NG tube CXRs.^3^^,^^13^ This gap in Scotland may also be present elsewhere in the United Kingdom. Given radiology residents’ interpretations are often decisive in initiating enteral nutrition delivery through NG tubes, this may contribute to patient safety risk.

### Resident low accuracy

Only 35.7% of medically qualified radiology residents (*N* = 10) correctly identified all 20 NG tube CXRs, collectively performing less well than observed in final year medical students who had undergone online training (58.2%; not compared statistically).^5^ The overall resident error rate was 8.9%, with errors including both misclassifying *correctly sited* tubes as mal-positioned and incorrectly interpreting tubes at the gastroesophageal junction as suitable for delivering enteral nutrition.

While attention has focused on preventing pulmonary misplacement of NG tubes (“never events”), this study reveals the hidden cost of inadequate training: patient discomfort, delayed nutrition/medication delivery, and unnecessary removal of correctly placed NG tubes, with knock-on financial and operational consequences for the health service.

### Impact of this study

Our previous study with senior medical students demonstrated that a free, interactive learning tool (30 NG tube CXRs) significantly improved interpretation accuracy.^5^ Students (*N* = 249) correctly interpreting all 20 CXRs rose from 7.6% to 58.2%, while those with at least one error fell from 51.9% to 27.8% (*P* < .001).

[The training module is accessible here: https://www.thestudentradiologist.co.uk/ng-tube-module/].

Following communication of the current study’s results, all Training Program Directors in Scotland implemented compulsory NG tube training for radiology residents, some adopting this learning module—an encouraging example of rapid translation of audit findings into practice.

### Wider medico-legal, educational, and training implications

A new competency-based training program for radiographer-led NG tube pathways has recently been launched in the United Kingdom.^11^ While promising, its validation and uptake by individual radiographers, departments, and governance bodies remain unclear.

Meanwhile, with 270 NG tube related “never events” reported between 2014 and 24, the legal and professional responsibility for safe interpretation of these examinations rests with the radiology service, with radiology residents frequently undertaking primary review.^6^

One proposed solution is shifting responsibility for all NG tube CXR interpretation to consultant (attending) radiologists. However, this is inefficient amid a severe workforce shortage and risks undermining educational responsibilities.^9^ Moreover, evidence suggests that targeted education can significantly improve performance.^5^

These issues deserve urgent attention, particularly in light of the December 2024 memorandum of understanding between UK regulatory, investigatory, and prosecutorial bodies.^14^ This guidance formalizes police investigations into suspected criminal actions by healthcare professionals resulting in death or life-altering harm, further raising the stakes for diagnostic safety.

### Potential limitations

#### Response rate

Despite repeated encouragement from Training Program Directors coupled with incentives, the response rate was suboptimal (15%) and could potentially limit the generalizability of these findings.^15^ Limited interest/time to engage with audit/quality improvement projects or a perception among radiology residents that the task (NG tube position on CXR) was either unimportant or already within their skill set could be some of the reasons.

Nevertheless, respondents were demographically representative of radiology residents across the whole of Scotland, including the mix of international graduates, across specialty-training years and direct-from-foundation entry into radiology^16^ (Table 1).

#### Self-selection bias

Participants may represent a more motivated or confident subgroup, which could explain higher accuracy in Scheme A (despite low response rate). However, Schemes B-D had similar response rates, making self-selection less likely to account for observed differences (Tables 1 and 2).

#### Test environment

CXRs were presented as TIFF images online (Google Form) with no measuring tools available—not as DICOM files on PACS workstations. This may have hindered identification of tube tips at the GOJ. However, these were the same images used in the medical student cohort after pilot testing to optimize viewing quality, suggesting this limitation did not substantially affect comparative interpretation.^5^

#### Generalizability

All participants were from Scottish training programs. While this, and the low response rate, could potentially limit generalizability of these findings, Scottish radiology residents perform comparably—and sometimes better—than peers in other UK regions (unpublished UKMED grey data, 2023). It is acknowledged that while training structures across the UK are broadly similar, this generalizability is not guaranteed.

#### Call to action

Despite potential limitations, this study highlights a need for UK-wide standardization of NG tube CXR interpretation training for radiology residents, and may support wider training for all clinicians responsible for the safe administration of enteral nutrition.

## Conclusion

This study highlights an important gap in radiology training, demonstrating that many radiology residents may not be adequately prepared to safely interpret NG tube position on chest X-ray. Given the significant clinical, safety, legal, and economic implications associated with misinterpretation, there is a need for mandatory, competency-based education across radiology residency training programs. Improving resident confidence and accuracy in NG tube interpretation is not just a curriculum issue—it is a patient safety imperative.

## Supplementary Material

tzag001_Supplementary_Data
