# Supplementary material for: Are radiology residents safe to report feeding nasogastric (NG) tubes on chest X-rays?
Source: BJR Open. 2026 Jan 10;8(1):tzag001. doi: 10.1093/bjro/tzag001 (PMC12872581; doi:10.1093/bjro/tzag001)

Appendix

You will be shown 20 NGT CXR. For each image, please state if each NG tube is appropriately sited for feeding.

Image 1: Is this NG tube appropriately sited for feeding? Yes / No

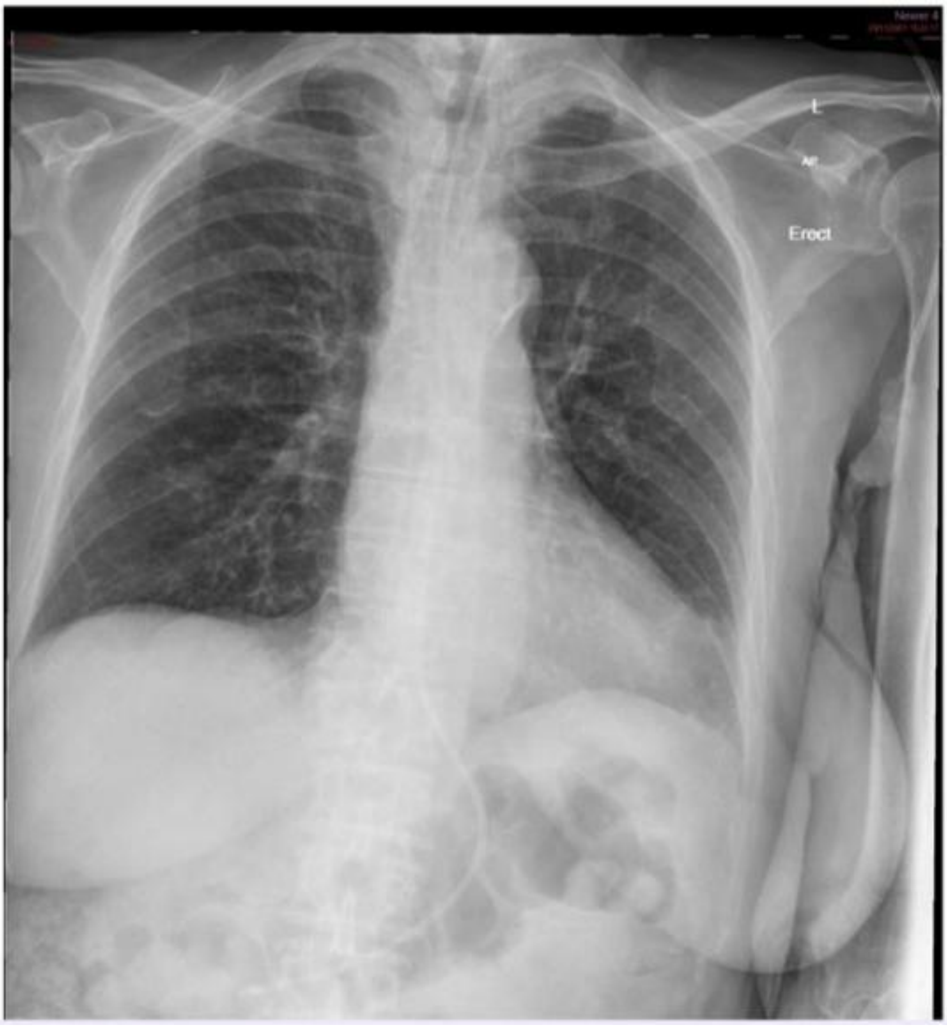

Supplement: tzag001_Supplementary_Data [file tzag001_supplementary_data.zip › Appendix NG.pdf]
