# Supplementary figures and images for: Are radiology residents safe to report feeding nasogastric (NG) tubes on chest X-rays?
Source: BJR Open. 2026 Jan 10;8(1):tzag001. doi: 10.1093/bjro/tzag001 (PMC12872581; doi:10.1093/bjro/tzag001)

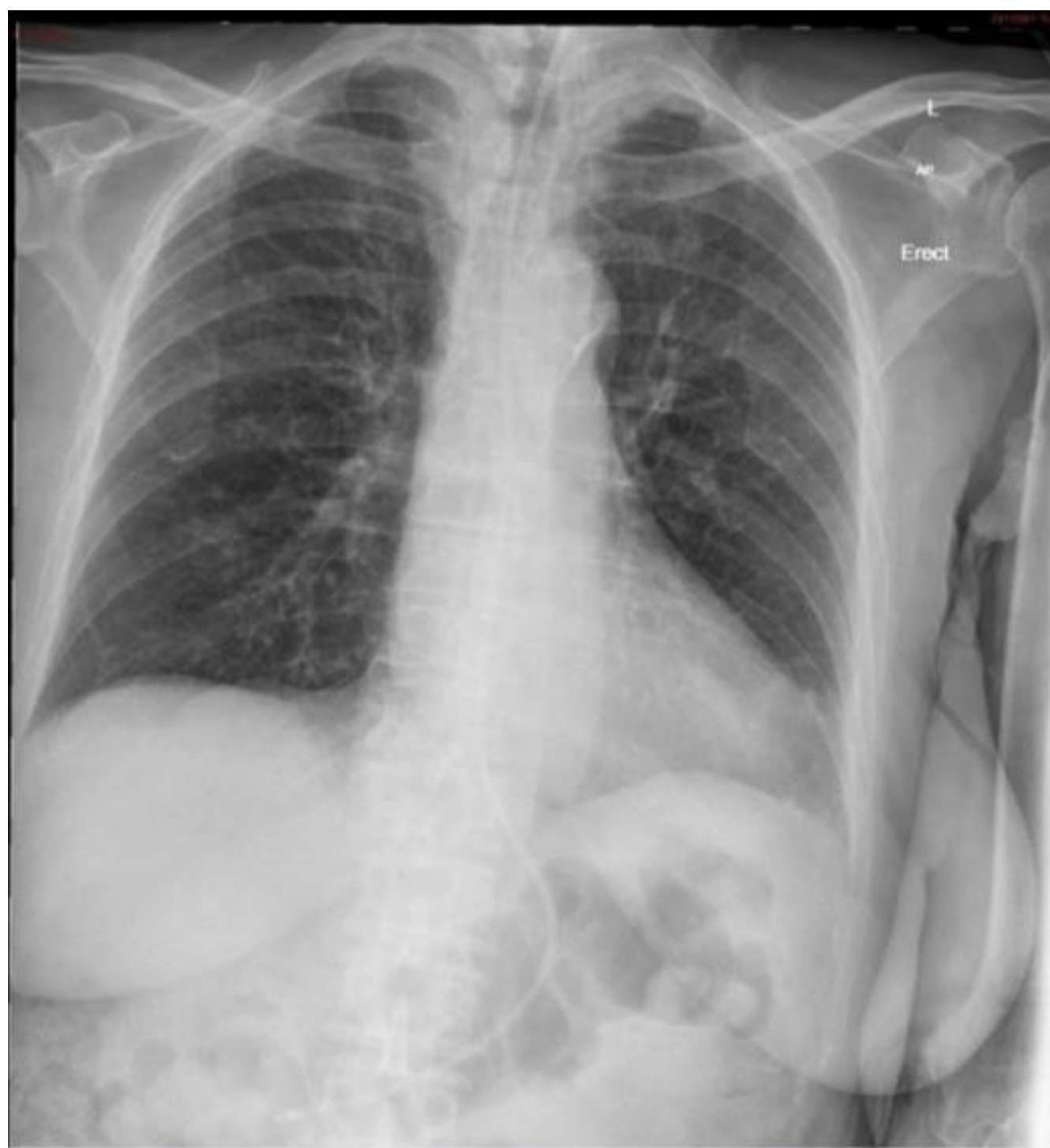

Supplement: tzag001_Supplementary_Data [file tzag001_supplementary_data.zip › Appendix figure.pdf]
